# 6-Meth­oxy-2-phenyl-4,4a,6,7,8,8a-hexa­hydro-2*H*-pyrano[3,2-*d*][1,3]dioxine-7,8-diyl bis­(4-methyl­benzene-1-sulfonate)

**DOI:** 10.1107/S1600536812006186

**Published:** 2012-02-17

**Authors:** James L. Wardell, Edward R. T. Tiekink

**Affiliations:** a Centro de Desenvolvimento Tecnológico em Saúde (CDTS), Fundação Oswaldo Cruz (FIOCRUZ), Casa Amarela, Campus de Manguinhos, Avenida Brasil 4365, 21040-900, Rio de Janeiro, RJ, Brazil; b Department of Chemistry, University of Malaya, 50603 Kuala Lumpur, Malaysia

## Abstract

In the title α-D-glucopyran­oside derivative, C_28_H_30_O_10_S_2_, each heterocyclic ring adopts a chair conformation. In the tri­substituted ring, the meth­oxy and one sulfonate group occupy axial positions, whereas the second sulfonate group occupies an axial position. The phenyl group on the other ring is in an equatorial position. In the crystal, supra­molecular chains propagating along [100] are formed through C—H⋯O and C—H⋯π inter­actions.

## Related literature

For the synthesis of the title compound, see: Brown *et al.* (1995 ▶); Whistler (1962 ▶). For the ^13^C NMR spectrum, see: Sugiyama *et al.* (1978 ▶).
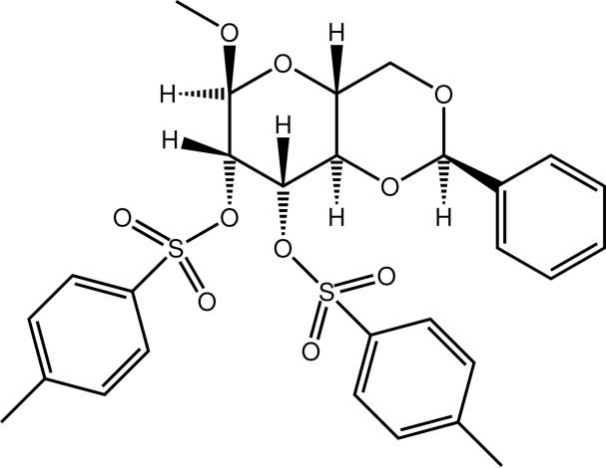


## Experimental

### 

#### Crystal data

C_28_H_30_O_10_S_2_
*M* *_r_* = 590.64
Orthorhombic, 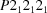

*a* = 5.7031 (16) Å
*b* = 17.020 (5) Å
*c* = 28.084 (8) Å
*V* = 2726.0 (14) Å^3^
*Z* = 4
Mo *K*α radiation
μ = 0.25 mm^−1^
*T* = 100 K
0.20 × 0.01 × 0.01 mm

#### Data collection

Rigaku Saturn724+ diffractometer
Absorption correction: multi-scan (*CrystalClear-SM Expert*; Rigaku, 2011 ▶) *T*_min_ = 0.747, *T*_max_ = 1.000
25991 measured reflections
6262 independent reflections
5326 reflections with *I* > 2σ(*I*)
*R*_int_ = 0.103

#### Refinement

*R*[*F* ^2^ > 2σ(*F* ^2^)] = 0.082
*wR*(*F* ^2^) = 0.147
*S* = 1.18
6262 reflections
364 parameters
H-atom parameters constrained
Δρ_max_ = 0.30 e Å^−3^
Δρ_min_ = −0.38 e Å^−3^
Absolute structure: Flack (1983 ▶), with 2656 Friedel pairs
Flack parameter: 0.21 (11)

### 

Data collection: *CrystalClear-SM Expert* (Rigaku, 2011 ▶); cell refinement: *CrystalClear-SM Expert*; data reduction: *CrystalClear-SM Expert*; program(s) used to solve structure: *SHELXS97* (Sheldrick, 2008 ▶); program(s) used to refine structure: *SHELXL97* (Sheldrick, 2008 ▶); molecular graphics: *ORTEP-3* (Farrugia, 1997 ▶) and *DIAMOND* (Brandenburg, 2006 ▶); software used to prepare material for publication: *publCIF* (Westrip, 2010 ▶).

## Supplementary Material

Crystal structure: contains datablock(s) global, I. DOI: 10.1107/S1600536812006186/hb6633sup1.cif

Structure factors: contains datablock(s) I. DOI: 10.1107/S1600536812006186/hb6633Isup2.hkl

Supplementary material file. DOI: 10.1107/S1600536812006186/hb6633Isup3.cml

Additional supplementary materials:  crystallographic information; 3D view; checkCIF report

## Figures and Tables

**Table 1:** Hydrogen-bond geometry (Å, °) *Cg*1 is the centroid of the C23–C28 phenyl ring.

*D*—H⋯*A*	*D*—H	H⋯*A*	*D*⋯*A*	*D*—H⋯*A*
C2—H2⋯O7^i^	1.00	2.59	3.548 (5)	162
C8—H8*B*⋯O1^i^	0.98	2.50	3.325 (6)	142
C10—H10⋯O7^i^	0.95	2.47	3.023 (6)	117
C20—H20⋯*Cg*1^i^	0.95	2.79	3.479 (5)	130

Symmetry code: (i) 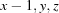.

**Fig. 1.**
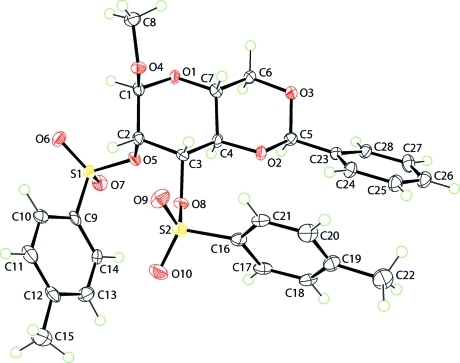
The molecular structure of (I) showing displacement ellipsoids at the 50% probability level.

**Fig. 2.**
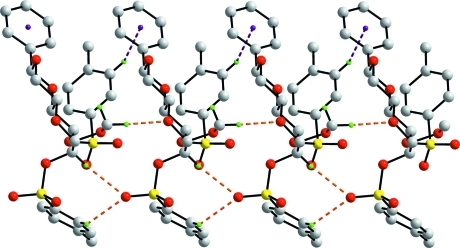
A view of the linear supramolecular chain along [100] in (I). The C—H···O and C—H···π interactions are shown as orange and purple dashed lines, respectively. H atoms not involved in the supramolecular interactions have been omitted for clarity.

**Fig. 3.**
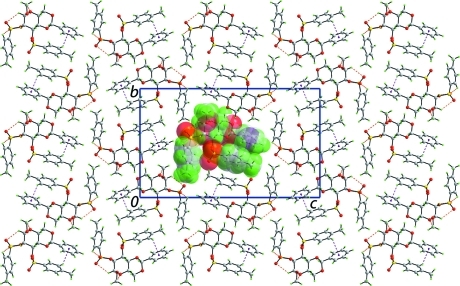
A view in projection down the a axis of the packing of supramolecular chains in (I). The C—H···O and C—H···π interactions are shown as orange and purple dashed lines, respectively. One supramolecular chain has been highlighted in space-filling mode.

## supplementary crystallographic information

### Comment

The title compound was prepared initially as a precursor for a series of 2-metallated derivatives of methyl 4,6-*O*-benzylidene-2-deoxy-α-*D*-altropyranosides (Brown *et al.*, 1995).

In (I), Fig. 1, both heterocyclic rings adopt a chair conformation. With respect to the fused heterocyclic rings, the phenyl and S2-sulfonate groups are approximately co-planar but, the S1-sulfonate group lies to one side, and the methoxy group to the other. In the O1-ring, the O4 and O5 substituents occupy axial positions whereas the O5 substituent is equatorial. In the O2-ring, the phenyl group is in an equatorial position.

In the crystal, C—H···O and C—H···π interactions link translationally related molecules into a supramolecular chain along [100], Fig. 2 and Table 1. Chains pack with no specific intermolecular interactions between them, Fig. 3.

### Experimental

The title compound was obtained from methyl 4,6-*O*-benzylidene-α-*D*-glucopyranoside and *p*-toluenesulfonyl chloride using a published procedure (Brown *et al.*, 1995; Whistler, 1962), m.p. 426–428 K. The ^13^C NMR spectrum was identical with that published (Sugiyama *et al.*, 1978).

### Refinement

The *C*-bound H atoms were geometrically placed (C—H = 0.95–1.00 Å) and refined as riding with *U*_iso_(H) = 1.2–1.5*U*_eq_(C). Owing to poor agreement, the (012) reflection was omitted from the final refinement. The absolute configuration of the molecule matches that of the α-*D*-glucopyranoside starting material.

### Crystal data

**Table:**

C_28_H_30_O_10_S_2_	*F*(000) = 1240
*M**_r_* = 590.64	*D*_x_ = 1.439 Mg m^−^^3^
Orthorhombic, *P*2_1_2_1_2_1_	Mo *K*α radiation, λ = 0.71073 Å
Hall symbol: P 2ac 2ab	Cell parameters from 9688 reflections
*a* = 5.7031 (16) Å	θ = 2.4–31.4°
*b* = 17.020 (5) Å	µ = 0.25 mm^−^^1^
*c* = 28.084 (8) Å	*T* = 100 K
*V* = 2726.0 (14) Å^3^	Needle, colourless
*Z* = 4	0.20 × 0.01 × 0.01 mm

### Data collection

**Table:**

Rigaku Saturn724+ diffractometer	6262 independent reflections
Radiation source: Rotating Anode	5326 reflections with *I* > 2σ(*I*)
Confocal monochromator	*R*_int_ = 0.103
Detector resolution: 28.5714 pixels mm^-1^	θ_max_ = 27.5°, θ_min_ = 2.5°
profile data from ω–scans	*h* = −7→5
Absorption correction: multi-scan (*CrystalClear*-SM Expert; Rigaku, 2011)	*k* = −22→22
*T*_min_ = 0.747, *T*_max_ = 1.000	*l* = −36→36
25991 measured reflections	

### Refinement

**Table:**

Refinement on *F*^2^	Secondary atom site location: difference Fourier map
Least-squares matrix: full	Hydrogen site location: inferred from neighbouring sites
*R*[*F*^2^ > 2σ(*F*^2^)] = 0.082	H-atom parameters constrained
*wR*(*F*^2^) = 0.147	*w* = 1/[σ^2^(*F*_o_^2^) + (0.0335*P*)^2^ + 1.481*P*] where *P* = (*F*_o_^2^ + 2*F*_c_^2^)/3
*S* = 1.18	(Δ/σ)_max_ < 0.001
6262 reflections	Δρ_max_ = 0.30 e Å^−^^3^
364 parameters	Δρ_min_ = −0.38 e Å^−^^3^
0 restraints	Absolute structure: Flack (1983), with 2656 Friedel pairs
Primary atom site location: structure-invariant direct methods	Flack parameter: 0.21 (11)

### Special details

**Table:**

Geometry. All s.u.'s (except the s.u. in the dihedral angle between two l.s. planes) are estimated using the full covariance matrix. The cell s.u.'s are taken into account individually in the estimation of s.u.'s in distances, angles and torsion angles; correlations between s.u.'s in cell parameters are only used when they are defined by crystal symmetry. An approximate (isotropic) treatment of cell s.u.'s is used for estimating s.u.'s involving l.s. planes.
Refinement. Refinement of *F*^2^ against ALL reflections. The weighted *R*-factor *wR* and goodness of fit *S* are based on *F*^2^, conventional *R*-factors *R* are based on *F*, with *F* set to zero for negative *F*^2^. The threshold expression of *F*^2^ > 2σ(*F*^2^) is used only for calculating *R*-factors(gt) *etc*. and is not relevant to the choice of reflections for refinement. *R*-factors based on *F*^2^ are statistically about twice as large as those based on *F*, and *R*-factors based on ALL data will be even larger.

### Fractional atomic coordinates and isotropic or equivalent isotropic displacement parameters (Å^2^)

**Table:**

	*x*	*y*	*z*	*U*_iso_*/*U*_eq_	
S1	0.5416 (2)	−0.05008 (7)	0.71777 (4)	0.0194 (3)	
S2	0.0901 (2)	0.09753 (6)	0.59013 (4)	0.0214 (3)	
O1	0.4150 (6)	−0.20508 (16)	0.61006 (9)	0.0164 (7)	
O2	0.4498 (5)	−0.05358 (16)	0.51713 (9)	0.0162 (6)	
O3	0.5946 (6)	−0.17301 (16)	0.48707 (10)	0.0186 (7)	
O4	0.0148 (5)	−0.18698 (16)	0.62785 (10)	0.0158 (7)	
O5	0.5053 (5)	−0.06571 (16)	0.66275 (10)	0.0168 (7)	
O6	0.4090 (6)	−0.10660 (18)	0.74447 (10)	0.0245 (8)	
O7	0.7922 (5)	−0.04735 (18)	0.72221 (11)	0.0232 (7)	
O8	0.3017 (6)	0.03820 (16)	0.59950 (10)	0.0187 (7)	
O9	−0.1278 (6)	0.05763 (18)	0.59845 (12)	0.0277 (8)	
O10	0.1498 (7)	0.16567 (17)	0.61702 (12)	0.0306 (9)	
C1	0.2469 (8)	−0.1719 (2)	0.64186 (15)	0.0156 (9)	
H1	0.2724	−0.1942	0.6744	0.019*	
C2	0.2700 (8)	−0.0829 (2)	0.64421 (15)	0.0154 (9)	
H2	0.1466	−0.0600	0.6653	0.019*	
C3	0.2614 (8)	−0.0455 (2)	0.59468 (15)	0.0161 (9)	
H3	0.1050	−0.0551	0.5797	0.019*	
C4	0.4517 (8)	−0.0828 (2)	0.56520 (14)	0.0149 (9)	
H4	0.6079	−0.0725	0.5801	0.018*	
C5	0.6340 (8)	−0.0903 (2)	0.49103 (15)	0.0167 (9)	
H5	0.7864	−0.0810	0.5077	0.020*	
C6	0.5940 (9)	−0.2098 (2)	0.53331 (14)	0.0190 (10)	
H6A	0.7491	−0.2034	0.5486	0.023*	
H6B	0.5617	−0.2667	0.5300	0.023*	
C7	0.4060 (8)	−0.1715 (2)	0.56347 (14)	0.0150 (9)	
H7	0.2486	−0.1816	0.5490	0.018*	
C8	−0.0436 (9)	−0.2694 (2)	0.63036 (16)	0.0232 (11)	
H8A	0.0214	−0.2967	0.6026	0.035*	
H8B	−0.2144	−0.2756	0.6307	0.035*	
H8C	0.0226	−0.2920	0.6595	0.035*	
C9	0.4247 (8)	0.0431 (3)	0.72699 (14)	0.0184 (9)	
C10	0.2123 (9)	0.0499 (3)	0.75107 (16)	0.0222 (10)	
H10	0.1389	0.0047	0.7643	0.027*	
C11	0.1086 (10)	0.1235 (3)	0.75561 (17)	0.0270 (11)	
H11	−0.0389	0.1282	0.7711	0.032*	
C12	0.2174 (9)	0.1902 (3)	0.73783 (16)	0.0240 (11)	
C13	0.4373 (9)	0.1825 (3)	0.71563 (17)	0.0266 (11)	
H13	0.5174	0.2281	0.7049	0.032*	
C14	0.5373 (9)	0.1099 (2)	0.70937 (15)	0.0217 (10)	
H14	0.6827	0.1052	0.6931	0.026*	
C15	0.1022 (11)	0.2697 (3)	0.74185 (19)	0.0381 (14)	
H15A	−0.0680	0.2638	0.7385	0.057*	
H15B	0.1614	0.3042	0.7166	0.057*	
H15C	0.1383	0.2926	0.7730	0.057*	
C16	0.1142 (9)	0.1205 (2)	0.52932 (15)	0.0184 (10)	
C17	0.3093 (9)	0.1617 (2)	0.51311 (17)	0.0212 (10)	
H17	0.4347	0.1732	0.5342	0.025*	
C18	0.3201 (9)	0.1857 (2)	0.46615 (17)	0.0227 (11)	
H18	0.4541	0.2133	0.4550	0.027*	
C19	0.1355 (10)	0.1698 (2)	0.43491 (16)	0.0246 (11)	
C20	−0.0548 (9)	0.1276 (3)	0.45155 (17)	0.0267 (11)	
H20	−0.1796	0.1157	0.4303	0.032*	
C21	−0.0680 (9)	0.1023 (2)	0.49803 (16)	0.0221 (10)	
H21	−0.1995	0.0729	0.5087	0.027*	
C22	0.1410 (11)	0.2005 (3)	0.38427 (18)	0.0373 (14)	
H22A	0.0745	0.1610	0.3628	0.056*	
H22B	0.3034	0.2114	0.3750	0.056*	
H22C	0.0485	0.2489	0.3822	0.056*	
C23	0.6480 (8)	−0.0578 (2)	0.44167 (14)	0.0161 (9)	
C24	0.4715 (8)	−0.0126 (2)	0.42182 (15)	0.0199 (10)	
H24	0.3351	−0.0008	0.4399	0.024*	
C25	0.4936 (9)	0.0159 (3)	0.37499 (16)	0.0244 (11)	
H25	0.3720	0.0469	0.3615	0.029*	
C26	0.6925 (9)	−0.0012 (3)	0.34847 (17)	0.0269 (12)	
H26	0.7071	0.0181	0.3169	0.032*	
C27	0.8701 (9)	−0.0465 (3)	0.36821 (16)	0.0238 (10)	
H27	1.0055	−0.0589	0.3500	0.029*	
C28	0.8494 (8)	−0.0738 (2)	0.41477 (15)	0.0188 (10)	
H28	0.9731	−0.1036	0.4284	0.023*	

### Atomic displacement parameters (Å^2^)

**Table:**

	*U*^11^	*U*^22^	*U*^33^	*U*^12^	*U*^13^	*U*^23^
S1	0.0230 (7)	0.0209 (5)	0.0143 (5)	−0.0029 (5)	−0.0011 (5)	−0.0003 (4)
S2	0.0258 (7)	0.0170 (5)	0.0214 (6)	0.0064 (5)	0.0024 (5)	−0.0009 (4)
O1	0.0212 (18)	0.0168 (14)	0.0112 (14)	0.0029 (14)	0.0029 (14)	0.0018 (11)
O2	0.0199 (17)	0.0173 (14)	0.0115 (13)	0.0039 (13)	0.0027 (13)	0.0018 (12)
O3	0.0251 (19)	0.0163 (14)	0.0146 (14)	0.0010 (14)	0.0036 (14)	−0.0011 (12)
O4	0.0112 (17)	0.0146 (14)	0.0216 (15)	−0.0015 (12)	−0.0001 (13)	−0.0007 (12)
O5	0.0167 (18)	0.0201 (15)	0.0136 (14)	−0.0015 (13)	−0.0009 (13)	−0.0051 (12)
O6	0.031 (2)	0.0244 (16)	0.0183 (16)	−0.0072 (16)	0.0004 (16)	0.0053 (13)
O7	0.0188 (18)	0.0279 (17)	0.0228 (17)	−0.0025 (15)	−0.0030 (15)	−0.0024 (15)
O8	0.0248 (18)	0.0114 (13)	0.0200 (16)	0.0011 (13)	0.0006 (14)	−0.0017 (12)
O9	0.0199 (19)	0.0273 (17)	0.036 (2)	0.0041 (15)	0.0104 (16)	0.0071 (15)
O10	0.045 (2)	0.0181 (16)	0.0287 (18)	0.0070 (16)	−0.0013 (18)	−0.0059 (14)
C1	0.014 (2)	0.020 (2)	0.013 (2)	−0.0005 (18)	0.0020 (18)	−0.0021 (17)
C2	0.013 (2)	0.019 (2)	0.014 (2)	−0.0028 (18)	0.0025 (18)	−0.0013 (17)
C3	0.022 (3)	0.0128 (19)	0.014 (2)	−0.0015 (18)	−0.0005 (19)	−0.0025 (17)
C4	0.018 (2)	0.0164 (19)	0.0107 (19)	0.0029 (18)	0.0004 (18)	0.0026 (15)
C5	0.016 (3)	0.0163 (19)	0.018 (2)	0.0004 (18)	0.0011 (19)	0.0013 (17)
C6	0.027 (3)	0.0151 (19)	0.015 (2)	0.001 (2)	0.001 (2)	0.0022 (17)
C7	0.015 (2)	0.0171 (19)	0.0131 (19)	0.0035 (18)	−0.0048 (19)	−0.0006 (16)
C8	0.029 (3)	0.017 (2)	0.024 (2)	−0.003 (2)	−0.001 (2)	0.0026 (18)
C9	0.022 (3)	0.023 (2)	0.0101 (19)	−0.003 (2)	−0.0036 (19)	−0.0075 (17)
C10	0.025 (3)	0.021 (2)	0.021 (2)	−0.008 (2)	0.001 (2)	−0.0062 (19)
C11	0.021 (3)	0.033 (3)	0.027 (3)	−0.006 (2)	0.005 (2)	−0.013 (2)
C12	0.030 (3)	0.025 (2)	0.018 (2)	−0.002 (2)	−0.002 (2)	−0.011 (2)
C13	0.032 (3)	0.023 (2)	0.025 (2)	−0.007 (2)	0.000 (3)	0.002 (2)
C14	0.029 (3)	0.023 (2)	0.014 (2)	−0.004 (2)	0.008 (2)	−0.0013 (17)
C15	0.043 (4)	0.027 (3)	0.043 (3)	0.000 (3)	0.008 (3)	−0.006 (2)
C16	0.020 (3)	0.0128 (19)	0.022 (2)	0.0045 (18)	−0.001 (2)	0.0017 (17)
C17	0.020 (3)	0.014 (2)	0.030 (3)	−0.0032 (19)	−0.007 (2)	−0.0016 (19)
C18	0.026 (3)	0.013 (2)	0.029 (3)	−0.004 (2)	−0.001 (2)	0.0024 (18)
C19	0.033 (3)	0.018 (2)	0.023 (2)	0.007 (2)	−0.001 (2)	0.0048 (19)
C20	0.025 (3)	0.026 (2)	0.029 (3)	0.001 (2)	−0.011 (2)	0.002 (2)
C21	0.025 (3)	0.0129 (19)	0.028 (2)	−0.004 (2)	0.000 (2)	−0.0001 (18)
C22	0.051 (4)	0.029 (3)	0.032 (3)	0.016 (3)	−0.005 (3)	0.005 (2)
C23	0.020 (3)	0.0150 (19)	0.014 (2)	0.0004 (18)	0.0030 (19)	0.0003 (17)
C24	0.018 (3)	0.023 (2)	0.018 (2)	−0.0002 (19)	0.004 (2)	0.0030 (17)
C25	0.028 (3)	0.027 (2)	0.018 (2)	−0.004 (2)	−0.005 (2)	0.0035 (19)
C26	0.032 (3)	0.030 (2)	0.019 (2)	−0.014 (2)	−0.004 (2)	0.004 (2)
C27	0.021 (3)	0.034 (3)	0.017 (2)	−0.002 (2)	0.004 (2)	−0.002 (2)
C28	0.019 (3)	0.021 (2)	0.016 (2)	−0.0050 (19)	0.001 (2)	0.0023 (17)

### Geometric parameters (Å, º)

**Table:**

S1—O7	1.435 (3)	C10—C11	1.392 (6)
S1—O6	1.435 (3)	C10—H10	0.9500
S1—O5	1.581 (3)	C11—C12	1.386 (7)
S1—C9	1.740 (4)	C11—H11	0.9500
S2—O10	1.425 (3)	C12—C13	1.406 (7)
S2—O9	1.435 (3)	C12—C15	1.508 (6)
S2—O8	1.595 (3)	C13—C14	1.372 (6)
S2—C16	1.757 (4)	C13—H13	0.9500
O1—C1	1.427 (5)	C14—H14	0.9500
O1—C7	1.429 (4)	C15—H15A	0.9800
O2—C5	1.426 (5)	C15—H15B	0.9800
O2—C4	1.439 (4)	C15—H15C	0.9800
O3—C5	1.429 (5)	C16—C17	1.392 (6)
O3—C6	1.442 (5)	C16—C21	1.396 (6)
O4—C1	1.404 (5)	C17—C18	1.382 (6)
O4—C8	1.444 (5)	C17—H17	0.9500
O5—C2	1.469 (5)	C18—C19	1.397 (7)
O8—C3	1.449 (4)	C18—H18	0.9500
C1—C2	1.522 (5)	C19—C20	1.383 (7)
C1—H1	1.0000	C19—C22	1.515 (6)
C2—C3	1.531 (6)	C20—C21	1.377 (6)
C2—H2	1.0000	C20—H20	0.9500
C3—C4	1.505 (6)	C21—H21	0.9500
C3—H3	1.0000	C22—H22A	0.9800
C4—C7	1.532 (5)	C22—H22B	0.9800
C4—H4	1.0000	C22—H22C	0.9800
C5—C23	1.495 (5)	C23—C24	1.384 (6)
C5—H5	1.0000	C23—C28	1.402 (6)
C6—C7	1.514 (6)	C24—C25	1.407 (6)
C6—H6A	0.9900	C24—H24	0.9500
C6—H6B	0.9900	C25—C26	1.388 (7)
C7—H7	1.0000	C25—H25	0.9500
C8—H8A	0.9800	C26—C27	1.389 (7)
C8—H8B	0.9800	C26—H26	0.9500
C8—H8C	0.9800	C27—C28	1.393 (6)
C9—C10	1.392 (6)	C27—H27	0.9500
C9—C14	1.397 (6)	C28—H28	0.9500
			
O7—S1—O6	120.1 (2)	C10—C9—C14	120.3 (4)
O7—S1—O5	102.74 (18)	C10—C9—S1	118.8 (4)
O6—S1—O5	109.21 (18)	C14—C9—S1	120.9 (4)
O7—S1—C9	109.8 (2)	C9—C10—C11	119.3 (4)
O6—S1—C9	109.3 (2)	C9—C10—H10	120.4
O5—S1—C9	104.40 (18)	C11—C10—H10	120.4
O10—S2—O9	120.4 (2)	C12—C11—C10	120.9 (5)
O10—S2—O8	104.30 (19)	C12—C11—H11	119.5
O9—S2—O8	109.19 (17)	C10—C11—H11	119.5
O10—S2—C16	108.4 (2)	C11—C12—C13	118.9 (5)
O9—S2—C16	109.3 (2)	C11—C12—C15	120.8 (5)
O8—S2—C16	103.98 (19)	C13—C12—C15	120.3 (5)
C1—O1—C7	113.0 (3)	C14—C13—C12	120.7 (4)
C5—O2—C4	109.0 (3)	C14—C13—H13	119.6
C5—O3—C6	111.0 (3)	C12—C13—H13	119.6
C1—O4—C8	112.4 (3)	C13—C14—C9	119.8 (4)
C2—O5—S1	120.0 (3)	C13—C14—H14	120.1
C3—O8—S2	119.1 (3)	C9—C14—H14	120.1
O4—C1—O1	112.7 (3)	C12—C15—H15A	109.5
O4—C1—C2	106.0 (3)	C12—C15—H15B	109.5
O1—C1—C2	111.3 (3)	H15A—C15—H15B	109.5
O4—C1—H1	108.9	C12—C15—H15C	109.5
O1—C1—H1	108.9	H15A—C15—H15C	109.5
C2—C1—H1	108.9	H15B—C15—H15C	109.5
O5—C2—C1	107.0 (3)	C17—C16—C21	120.1 (4)
O5—C2—C3	105.6 (3)	C17—C16—S2	119.5 (4)
C1—C2—C3	111.9 (3)	C21—C16—S2	120.3 (4)
O5—C2—H2	110.7	C18—C17—C16	119.8 (5)
C1—C2—H2	110.7	C18—C17—H17	120.1
C3—C2—H2	110.7	C16—C17—H17	120.1
O8—C3—C4	110.6 (3)	C17—C18—C19	120.5 (5)
O8—C3—C2	108.6 (3)	C17—C18—H18	119.7
C4—C3—C2	107.5 (3)	C19—C18—H18	119.7
O8—C3—H3	110.0	C20—C19—C18	118.7 (4)
C4—C3—H3	110.0	C20—C19—C22	120.8 (5)
C2—C3—H3	110.0	C18—C19—C22	120.5 (5)
O2—C4—C3	111.4 (3)	C21—C20—C19	121.7 (5)
O2—C4—C7	108.0 (3)	C21—C20—H20	119.1
C3—C4—C7	108.1 (4)	C19—C20—H20	119.1
O2—C4—H4	109.8	C20—C21—C16	119.1 (4)
C3—C4—H4	109.8	C20—C21—H21	120.4
C7—C4—H4	109.8	C16—C21—H21	120.4
O2—C5—O3	110.9 (3)	C19—C22—H22A	109.5
O2—C5—C23	110.7 (3)	C19—C22—H22B	109.5
O3—C5—C23	107.5 (3)	H22A—C22—H22B	109.5
O2—C5—H5	109.2	C19—C22—H22C	109.5
O3—C5—H5	109.2	H22A—C22—H22C	109.5
C23—C5—H5	109.2	H22B—C22—H22C	109.5
O3—C6—C7	108.6 (3)	C24—C23—C28	119.2 (4)
O3—C6—H6A	110.0	C24—C23—C5	122.7 (4)
C7—C6—H6A	110.0	C28—C23—C5	118.1 (4)
O3—C6—H6B	110.0	C23—C24—C25	120.2 (4)
C7—C6—H6B	110.0	C23—C24—H24	119.9
H6A—C6—H6B	108.4	C25—C24—H24	119.9
O1—C7—C6	108.3 (3)	C26—C25—C24	120.1 (5)
O1—C7—C4	111.0 (3)	C26—C25—H25	119.9
C6—C7—C4	108.8 (4)	C24—C25—H25	119.9
O1—C7—H7	109.6	C25—C26—C27	119.9 (4)
C6—C7—H7	109.6	C25—C26—H26	120.1
C4—C7—H7	109.6	C27—C26—H26	120.1
O4—C8—H8A	109.5	C26—C27—C28	119.9 (5)
O4—C8—H8B	109.5	C26—C27—H27	120.1
H8A—C8—H8B	109.5	C28—C27—H27	120.1
O4—C8—H8C	109.5	C27—C28—C23	120.7 (4)
H8A—C8—H8C	109.5	C27—C28—H28	119.7
H8B—C8—H8C	109.5	C23—C28—H28	119.7
			
O7—S1—O5—C2	171.9 (3)	O5—S1—C9—C10	107.5 (3)
O6—S1—O5—C2	43.4 (3)	O7—S1—C9—C14	39.0 (4)
C9—S1—O5—C2	−73.4 (3)	O6—S1—C9—C14	172.7 (4)
O10—S2—O8—C3	155.0 (3)	O5—S1—C9—C14	−70.6 (4)
O9—S2—O8—C3	25.1 (3)	C14—C9—C10—C11	2.7 (7)
C16—S2—O8—C3	−91.5 (3)	S1—C9—C10—C11	−175.3 (4)
C8—O4—C1—O1	66.1 (4)	C9—C10—C11—C12	−2.0 (7)
C8—O4—C1—C2	−172.0 (3)	C10—C11—C12—C13	−1.0 (7)
C7—O1—C1—O4	64.3 (4)	C10—C11—C12—C15	178.7 (5)
C7—O1—C1—C2	−54.6 (5)	C11—C12—C13—C14	3.4 (7)
S1—O5—C2—C1	−95.2 (3)	C15—C12—C13—C14	−176.2 (5)
S1—O5—C2—C3	145.5 (3)	C12—C13—C14—C9	−2.7 (7)
O4—C1—C2—O5	175.3 (3)	C10—C9—C14—C13	−0.4 (7)
O1—C1—C2—O5	−61.9 (4)	S1—C9—C14—C13	177.7 (4)
O4—C1—C2—C3	−69.6 (4)	O10—S2—C16—C17	45.1 (4)
O1—C1—C2—C3	53.2 (5)	O9—S2—C16—C17	178.1 (3)
S2—O8—C3—C4	132.3 (3)	O8—S2—C16—C17	−65.4 (4)
S2—O8—C3—C2	−109.9 (3)	O10—S2—C16—C21	−130.6 (4)
O5—C2—C3—O8	−59.8 (4)	O9—S2—C16—C21	2.4 (4)
C1—C2—C3—O8	−175.8 (3)	O8—S2—C16—C21	118.9 (3)
O5—C2—C3—C4	59.9 (4)	C21—C16—C17—C18	1.2 (6)
C1—C2—C3—C4	−56.1 (5)	S2—C16—C17—C18	−174.5 (3)
C5—O2—C4—C3	179.6 (3)	C16—C17—C18—C19	0.7 (7)
C5—O2—C4—C7	−61.9 (4)	C17—C18—C19—C20	−1.8 (7)
O8—C3—C4—O2	−64.2 (4)	C17—C18—C19—C22	176.3 (4)
C2—C3—C4—O2	177.4 (3)	C18—C19—C20—C21	1.1 (7)
O8—C3—C4—C7	177.3 (3)	C22—C19—C20—C21	−176.9 (4)
C2—C3—C4—C7	58.8 (4)	C19—C20—C21—C16	0.7 (7)
C4—O2—C5—O3	64.4 (4)	C17—C16—C21—C20	−1.8 (6)
C4—O2—C5—C23	−176.4 (3)	S2—C16—C21—C20	173.8 (3)
C6—O3—C5—O2	−62.6 (5)	O2—C5—C23—C24	−13.9 (6)
C6—O3—C5—C23	176.3 (4)	O3—C5—C23—C24	107.3 (4)
C5—O3—C6—C7	57.9 (5)	O2—C5—C23—C28	165.9 (4)
C1—O1—C7—C6	178.9 (3)	O3—C5—C23—C28	−72.9 (5)
C1—O1—C7—C4	59.6 (5)	C28—C23—C24—C25	0.8 (6)
O3—C6—C7—O1	−176.8 (3)	C5—C23—C24—C25	−179.4 (4)
O3—C6—C7—C4	−56.0 (4)	C23—C24—C25—C26	0.0 (7)
O2—C4—C7—O1	177.6 (3)	C24—C25—C26—C27	0.0 (7)
C3—C4—C7—O1	−61.7 (5)	C25—C26—C27—C28	−0.8 (7)
O2—C4—C7—C6	58.5 (4)	C26—C27—C28—C23	1.7 (7)
C3—C4—C7—C6	179.2 (3)	C24—C23—C28—C27	−1.6 (6)
O7—S1—C9—C10	−143.0 (3)	C5—C23—C28—C27	178.6 (4)
O6—S1—C9—C10	−9.3 (4)		

### Hydrogen-bond geometry (Å, º)

Cg1 is the centroid of the C23–C28 phenyl ring.

**Table:**

*D*—H···*A*	*D*—H	H···*A*	*D*···*A*	*D*—H···*A*
C2—H2···O7^i^	1.00	2.59	3.548 (5)	162
C8—H8*B*···O1^i^	0.98	2.50	3.325 (6)	142
C10—H10···O7^i^	0.95	2.47	3.023 (6)	117
C20—H20···*Cg*1^i^	0.95	2.79	3.479 (5)	130

Symmetry code: (i) *x*−1, *y*, *z*.
